# Genuine and Spurious Phase Synchronization Strengths during Consciousness and General Anesthesia

**DOI:** 10.1371/journal.pone.0046313

**Published:** 2012-10-02

**Authors:** UnCheol Lee, HeonSoo Lee, Markus Müller, Gyu-Jeong Noh, George A. Mashour

**Affiliations:** 1 Division of Neuroanesthesiology, Department of Anesthesiology, University of Michigan Medical School, Ann Arbor, Michigan, United States of America; 2 Department of Physics, POSTECH, Pohang, South Korea; 3 Facultad de Ciencias, Universidad Autónoma del Estado de Morelos, Centro Internacional de Ciencias, Universidad Nacional Autónoma de México, Cuernavaca, Morelos, México; 4 Department of Clinical Pharmacology and Therapeutics, Department of Anesthesiology and Pain Medicine, Asan Medical Center, University of Ulsan College of Medicine, Seoul, Korea; 5 Neuroscience Graduate Program, University of Michigan, Ann Arbor, Michigan, United States of America; University of British Columbia, Canada

## Abstract

Spectral content in a physiological dataset of finite size has the potential to produce spurious measures of coherence. This is especially true for electroencephalography (EEG) during general anesthesia because of the significant alteration of the power spectrum. In this study we quantitatively evaluated the genuine and spurious phase synchronization strength (PSS) of EEG during consciousness, general anesthesia, and recovery. A computational approach based on the randomized data method was used for evaluating genuine and spurious PSS. The validity of the method was tested with a simulated dataset. We applied this method to the EEG of normal subjects undergoing general anesthesia and investigated the finite size effects of EEG references, data length and spectral content on phase synchronization. The most influential factor for genuine PSS was the type of EEG reference; the most influential factor for spurious PSS was the spectral content. Genuine and spurious PSS showed characteristic temporal patterns for each frequency band across consciousness and anesthesia. Simultaneous measurement of both genuine and spurious PSS during general anesthesia is necessary in order to avoid incorrect interpretations regarding states of consciousness.

## Introduction

The administration of general anesthetics results in dramatic changes in behavioral state, which is accompanied by changes of functional connectivity and information integration capacity in the brain [1]–[6]. Phase synchronization is thought to be one potential candidate for integrating neuronal sources distributed across specialized brain areas [7]–[11]. As such, it has been widely used to quantify the underlying mechanism of large scale integration in the brain based on neurophysiologic recordings. Although a valuable technique, several limitations in the evaluation of EEG phase synchronization have also been noted [12]–[15]. Limitations such as volume conduction, the active reference electrode and the finite size effect due to spectral content are fundamental problems causing spurious or distorted coherence measures of brain activities. Several methods have been introduced to minimize these problems, but the difficulty persists. Volume conduction, which results when a neural source is observable at more than one electrode, could be attenuated by baseline correction and the method of localizing activities [12] [16]. A quiet reference electrode and careful interpretation have been suggested for the active reference electrode problem. Finally, the randomized data test or bootstrap method have been suggested for the finite size effect due to spectral content [12] [14]–[15]. However, the effects of each of these limitations on the degree of spurious phase synchronization have not been systematically quantified.

Random or spurious correlations within multivariate data sets have been studied by random matrix theory (RMT), in which the random part of a correlation can be predicted based on the analytic results obtained from random matrix ensembles. Genuine correlation can then be estimated based on significant deviation from the analytic RMT predictions. Information about the correlation structure of the multivariate data set is imprinted into the structures of a correlation matrix [17]–[22]. By decomposing the linear correlation matrix of multichannel EEG data into principle structures and comparing them with randomized data, the spurious and genuine correlation structures have been estimated [23]. Genuine correlations were defined as the correlation values that were above and beyond those found in the randomized dataset. It is known that spectral content dominated by lower frequencies is associated with larger spurious correlation [23]. As such, the dramatic shift toward lower frequency spectral contents during general anesthesia may seriously distort the coherence estimation. Despite the known potential for distorted coherence measures, there has been relatively little attention to quantitative assessment of the phenomenon [24], [25].

In the current study, indices of genuine, spurious and total phase synchronization strength (PSS) were quantified in the EEG of conscious and anesthetized human volunteers. First, simulated data based on the N-tori model were used to test the validity of genuine PSS measures. This model simulated multichannel oscillations and dynamic state transitions together with the modulation of phase coherence and the dominant spectral content. Second, in order to quantify spurious elements, we used randomized data with the same spectral content as the original EEG but with zero coherence. Third, the following were investigated as different sources of spurious phase synchronization: four types of EEG reference, six frequency bands and different data lengths. Finally, we investigated the temporal evolution pattern of the genuine, spurious and total PSS during general anesthesia.

## Results

### Genuine PSS in a Simulated EEG Time Series

**Figure 1:**
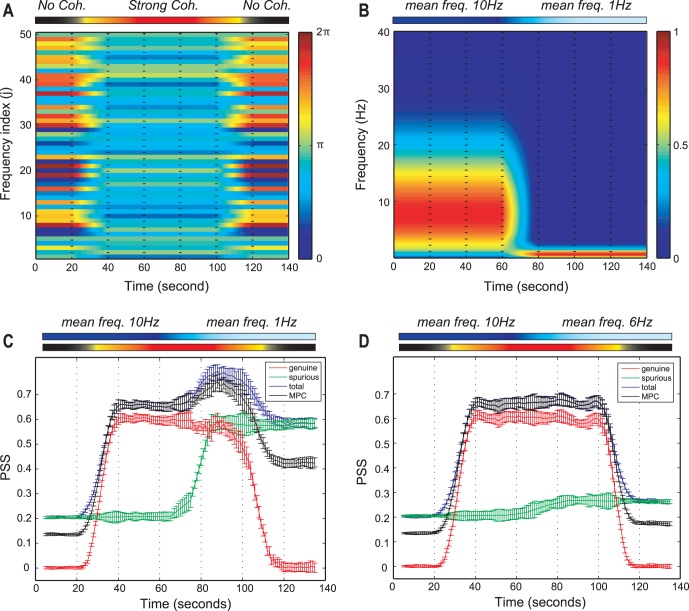
The test of the three PSS indices using the simulated EEG time series. (a) The phases of 50 sine waves for a simulated EEG time series are presented. The non-coherent (0–20 s and 120–140 s) and coherent (40–100 s) phases are given to the simulation, with two linear state transitions (20–40 s and 100–120 s). The color bar on the right side indicates a radian within 0 to 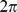 and the frequency index (j) reflects the indices of first 50 sine waves among 5,000 (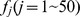 of Eq.4) for visualization. (b) The time-frequency plot of a simulated EEG time series is presented. The two time periods have two different dominant frequencies (10 Hz for the first half period and 1 Hz for the other half). The state transition between two dominant frequencies takes place during 60–80 s. The color bar on the right side denotes the probability density for the frequencies. (c) The total, spurious, genuine PSS and the mean phase coherence (MPC) for the two mean frequencies (10 Hz and 1 Hz) are presented. The genuine PSS (red) correlates well with the modulated coherence, while the spurious PSS (green) correlates with the mean frequency. The MPC has non-zero values in non-coherent periods, which appears amplified in the lower mean frequency period (1 Hz). (d) Same as (c), but with means frequencies of 10 and 6 Hz, respectively. The spurious PSS was significantly reduced with the mean frequency of 6 Hz, but was still larger than that of 10 Hz. The error bars denote the standard deviation. The upper bars in (a)–(d) indicate the states of coherence and different mean frequencies across EEG epochs.

To test the validity of the genuine PSS, 20 EEG time series simulated by the N-tori model were used (Figure 1). In the model, the phase coherence and the mean frequency of 20 time series were modulated in different ways across seven periods (see Methods). Figure 1 (a) presents the EEG epochs of three different levels of coherence: no coherence (0–20 s and 120–140 s), coherence (40–100 s), and transition (20–40 s and 100–120 s). Figure 1(b) presents the EEG epochs of two different mean frequencies: 10 Hz (0–60 s), 1 Hz (80–140 s), and transition between two mean frequencies (60–80 s). Figure 1 (c) and (d) demonstrated the effect of mean frequencies (1, 6 and 10 Hz) and the relationship of genuine, spurious and total PSS with the true coherence given in the simulation.

In the first and the last 20 s epochs for each EEG time series simulation, the partial phases 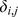 was chosen from a uniform distribution ranging from 0 to 2π for no coherency. On the other hand, during the 40–100 s epoch, the phases for the corresponding frequencies were chosen from a normal distribution with mean phase (i.e., 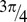). Spurious PSS quantifies the PSS that is falsely produced by finite size and certain spectral contents of the data. Genuine PSS quantifies the phase synchronization strength that deviates from that of the randomized data set. Total PSS reflects all PSS (spurious and genuine) contained in a given set of data.

Figure 1(c) demonstrates the increasing or decreasing patterns of genuine PSS at the two state transition periods and the large genuine PSS during 40–100 s (p<0.0001, F (6, 63) = 586, repeated measures one-way analysis of variance (ANOVA) with Tukey’s multiple comparison test). For the two zero-coherent periods (0–20 s and 120–140 s), the genuine PSS had a value of zero. After shifting the mean frequency from 10 Hz to 1 Hz during 60–120 s (Figure 1(b)), the spurious PSS was significantly increased (Figure 1(c)) (p<0.0001, F(6,63) = 6,448). Furthermore, the spurious PSS exactly reflected the change of the mean frequency during the period (60–80 s) (green line in Figure 1(c)). The total PSS was affected by the genuine and spurious components. The mean phase coherence, which is the average value over all pairs of 20 time series, showed a pattern that paralleled the total PSS. However, as expected, the mean phase coherence did not have zero-values in the two non-coherent periods (0–20 s and 120–140 s). Furthermore, mean phase coherence showed a larger non-zero value in the lower frequency dominant period (120–140 s). These data directly demonstrate the contamination of mean phase coherence measures by spurious coherence components.

In contrast, the spurious phase coherence components were significantly reduced as the mean frequency was increased. Figure 1 (d) shows the PSS indices and the mean phase coherence for the time series with two mean frequencies (10 Hz for 0–60 s and 6 Hz for 60–140 s). The spurious PSS had a certain non-zero value over the whole period, showing a tendency of a slight increase from 60 s after the mean frequency shifted toward 6 Hz. Because of the flat spurious PSS, the total PSS and the mean phase coherence showed a similar pattern with that of genuine PSS. However, the problem of a non-zero value in the non-coherent periods remained. It is also of note that the spurious phase coherence component was significantly amplified as the mean frequency was decreased below 6 Hz (Figure 1 (d), p<0.0001, F(6,63) = 736.5, repeated measures one-way ANOVA), which corresponds to the theta and delta rhythms of the EEG.

### Significant Change of Spectral Content during General Anesthesia

**Figure 2:**
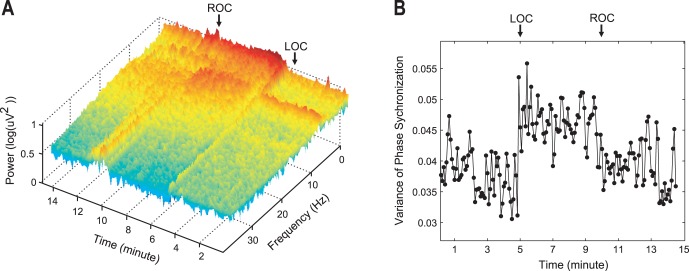
Example of changes of power spectrum and phase synchronization after anesthesia. (a) The averaged spectrogram of 21 EEG channels for a subject, and (b) the variance of phase synchronizations between 21 EEG channels for a subject during general anesthesia. This demonstrates the significant changes of the power spectrum and the variance of phase synchronization during general anesthesia. The loss of consciousness and the recovery of consciousness happen at 5 and 10 minutes.

Fig. 2 (a) demonstrates an example of the temporal evolution of power spectra over the entire frequency range during general anesthesia. The short-term Fourier transform method was applied to each 10 second long window for 21 EEG channels of a subject (Spectrogram, in Matlab toolbox). The averaged short-term Fourier transform over 21 EEG channels is shown in Figure 2(a). After induction of general anesthesia there was a large increase of lower frequency power as well as an increased variance of mean phase synchronizations over all pairs of EEG channels (Figure 2(b)). The increased variance implies that the structure of phase synchronizations among the 21 EEG channels became more complex in the anesthetized states and could produce various changes of PSS indices. The EEG simulation with N-tori predicts that the increased lower-frequency power in anesthetized state induces larger spurious phase synchronization components, which could inaccurately reflect the coherence of brain activities.

### Genuine, Spurious and Total PSS for Randomized Data

**Figure 3:**
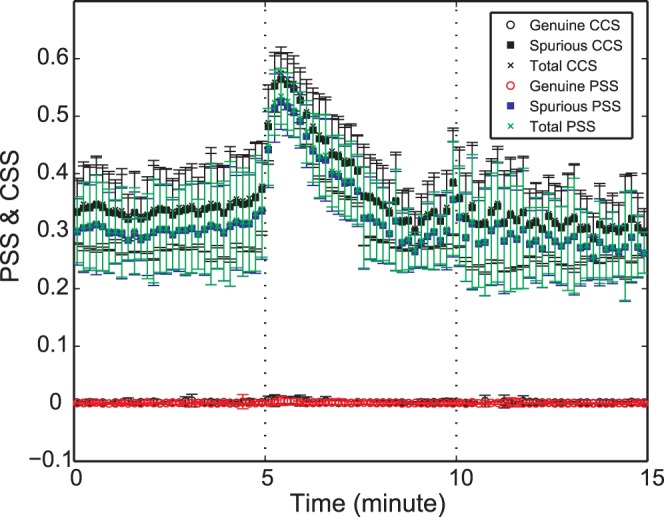
Spurious phase synchronization strength (PSS) during general anesthesia. The mean of genuine, spurious and total PSS over all randomized data sets generated from all subjects’ original EEG data are presented over time (the error bar denotes the standard deviation). The spurious PSS (blue square) and the spurious correlation strength by Pearson correlation coefficient (black square, denoted as “CCS”) were compared. (Vertical dotted lines: loss of consciousness and return of consciousness points, sequentially). The linear Pearson correlation and the phase synchronization produce a large spurious component after anesthesia.

The spurious PSS was estimated from randomized human EEG data in order to evaluate the influence of spectral shifts after general anesthesia that were predicted by the N-Tori model. Since the randomized data has the same power spectrum with that of the broadband EEG but with zero-coherence by randomization, we can selectively evaluate the effect of spectral shift on the spurious PSS index. The temporal patterns of the spurious, genuine and total PSS were examined by the moving window method to investigate the effect of dynamic spectral change during state transitions (around loss of consciousness [LOC] and return of consciousness [ROC], at 5 and 10 minutes on the time axis, respectively). The means and standard deviations over 20 EEG data sets are presented in Figure 3, which demonstrates significant increases of the spurious PSS (blue dots) (p<0.0001, F(6,63) = 57.58) and increases in total PSS (green dots) (p<0.0001, F(6,63) = 59.93) after LOC. The spurious and total PSS gradually decreased down to the level of the baseline waking state, while the genuine PSS was nearly zero during the whole experiment. The total PSS (green) has the same temporal pattern as the spurious PSS (blue) because of the zero genuine PSS (red) over the entire experiment. This indicates that the total PSS was significantly influenced by the spurious phase synchronization, primarily induced by the finite size effect of specific spectral contents during general anesthesia.

Two measures, phase synchronization (nonlinear) and Pearson correlation coefficient (linear), were compared to assess their vulnerability to the finite size effect for this data set. For the genuine, spurious and total linear correlation strengths, the mean phase coherence in the definitions of genuine, spurious and total PSS was replaced with Pearson correlation coefficient 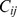
between two EEG datasets (*i,j*) [18]. In Figure 3, the spurious linear Pearson correlation strength (black square) was slightly larger than the spurious PSS (blue square), but not significantly different.

### Finite Size Effects of Data Length, Frequency Band and Reference for Empirical EEG Data

**Figure 4:**
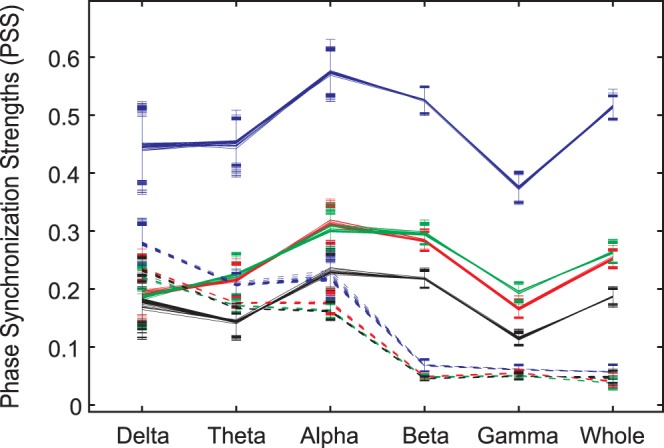
The effects of EEG reference, data length and frequency band on genuine and spurious phase synchronization strength (PSS). Each symbol denotes the mean genuine PSS (solid lines) or the mean spurious PSS (dotted lines) for the combination of data length, type of EEG reference and frequency band. The EEG reference mainly affects genuine PSS. Each color denotes a type of EEG reference (blue: unipolar reference (A2); green: longitudinal bipolar; red: common averaged reference; and black: transverse bipolar). The unipolar reference has the largest genuine PSS over all frequency bands. By contrast, the frequency band mainly influences spurious PSS. Lower frequency bands have larger spurious PSS over all types of EEG references. The error bars denote the standard deviations over the EEG data sets for 27 data lengths (from 5 to 60 seconds, with 2 second intervals). Six frequency bands were studied: delta band (0.5–4 Hz), theta band (4–8 Hz), alpha band (8–13 Hz), beta band (13–25 Hz), gamma band (25–55 Hz) and whole band (0.5–55 Hz).

The finite size effects induced by data length, EEG reference and frequency band were investigated using the EEG in the baseline conscious state (Figure 4). The EEG (5minutes) was segmented into different data lengths, from 5 second- to 60 second-long windows with a 2 second interval. The means of genuine and spurious PSS were calculated over 5 minute-long EEG for each data length. There was no significant dependence of the genuine and spurious PSS on these data lengths.

In contrast to data length, the EEG reference had a significant effect on the genuine and spurious PSS (Figure 4). The unipolar reference (A2) had a relatively larger genuine PSS (blue solid line) in all frequency bands, while the transverse bipolar reference had the lowest genuine PSS (black solid line). The common reference and the longitudinal bipolar reference (red and green solid lines, respectively) had similar genuine PSS values over all frequency bands.

The frequency band demonstrated the most significant effect on spurious PSS. The lower frequency bands (delta, theta and alpha) had a relatively larger spurious PSS, compared to the higher frequency bands (beta, gamma). In general, the unipolar reference produces relatively large coherence artificially.

In this study, there was a biased electrode distribution (primarily frontal and parietal regions), which could produce biased PSS with distant clustered electrodes. As such, it is difficult to distinguish the effect of volume conduction and the effect of biased electrode distribution on the PSS indices in this data. In the analysis of anesthesia EEG, we assumed that the volume conduction was preserved across states and therefore focused on the increase or decrease of PSS indices relative to those of the baseline state.

### The Temporal Patterns of Genuine, Spurious and Total PSS during General Anesthesia

**Figure 5:**
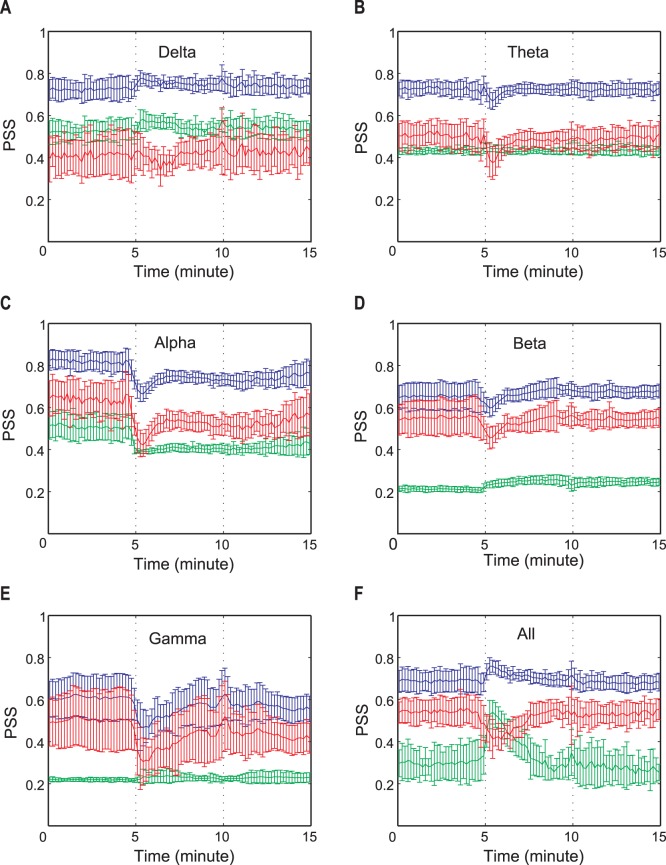
The temporal evolution of genuine, spurious and total PSS during general anesthesia for the six frequency bands. The mean genuine, spurious and total PSS are denoted by different colors (red: genuine PSS; green: spurious PSS; and blue: total PSS). The error bar denotes the standard deviations of genuine, spurious and total PSS values over all EEG datasets. The vertical dotted lines indicate the loss of consciousness and recovery of consciousness, sequentially. Six frequency bands were studied: delta band (0.5–4 Hz), theta band (4–8 Hz), alpha band (8–13 Hz), beta band (13–25 Hz), gamma band (25–55 Hz) and whole band (0.5–55 Hz).

The three PSS indices were applied to EEG data recorded during baseline conscious, unconscious (loss of consciousness, LOC) and recovery states (return of consciousness, ROC). Each state consists of a 5 minute-long EEG epoch. Figure 5(a)–(f) demonstrates the temporal patterns of genuine, spurious and total PSS for the six frequency bands across different states of consciousness. For clarity of statistical analysis, each state was separated into two equal sub-periods (baseline waking state: B1 and B2, anesthetized state: A1 and A2, and recovery state: R1 and R2). The results of the statistical tests for the changes of the three PSS indices are presented in Table 1. In Figure 5, the means and standard deviations of the three PSS indices over all EEG datasets are presented. The EEG of different frequency bands had distinct temporal patterns for each PSS. Here, the temporal patterns for the unipolar EEG reference were presented; the other EEG references showed a qualitatively similar behavior.

**Table 1:** The statistical test for genuine (g), spurious(s) and total (t) PSS indices**.**

	Delta			Theta			Alpha			Beta			Gamma			Whole		
	*g*	*s*	*t*	*g*	*s*	*t*	*g*	*s*	*t*	*g*	*s*	*t*	*g*	*s*	*t*	*g*	*s*	*t*
B1/B2																		
B1/**A1**		***	***	***		*	***	***	***	*	***		***		***	***	***	***
B1/A2			**				***	***	***		***	*						
B1/R1		**	*				***	***	***		***							
B1/R2							***	***	***		***		*					
B2/**A1**		***	***	***			***	***	***	*	***		***		***	***	***	***
B2/A2			*				***	***	***		***	*						
B2/R1							***	***	***		***							
B2/R2							***	***	***		***						*	
**A1/**A2		**		**						*		**	*		*	***	***	**
**A1**/R1		*		**						*		*	**		**	***	***	***
**A1**/R2		***	*	***			**		**			*				***	***	***
A2/R1																		
A2/R2																		

Tukey’s multiple comparison test with repeated one-way ANOVA.

*** p<0.0001,

** p<0.01,

* p<0.05.

Each period was separated into two sub-periods. Baseline conscious states: B1 and B2, Anesthetized states: A1 and A2, Recovery states: R1 and R2. The number of states is 6, and the number of each PSS index at each state is 20. The comparison to A1 is highlighted with bold font.

Repeated measures one-way ANOVA with Tukey’s multiple comparison test was applied to the six sub-states for each frequency band in the 20 EEG datasets (Table 1). The genuine PSS of the delta band was preserved across states (P>0.05), while the spurious PSS was significantly increased in A1 (p<0.0001). By contrast, no significant change of spurious PSS was found at A1 in the theta and gamma bands, but their genuine PSS were decreased (p<0.0001 and p<0.05). For the alpha band, the largest decreases took place in the three PSS indices after anesthesia. For the gamma band, the genuine PSS was decreased (p<0.0001) without significant change of spurious PSS. For the whole EEG band, the genuine PSS was reduced (p<0.0001), while the spurious PSS was significantly increased after LOC (p<0.0001).

Regarding the temporal evolution pattern, the genuine PSS was decreased after LOC in most frequency bands except the delta band. The decreased genuine PSS level of the alpha band was maintained until the end of recording, while the genuine PSS for the theta, gamma and whole frequency bands recovered in a short time. The alpha and gamma bands showed relatively large decreases of genuine PSS.

Regarding the spurious PSS, the delta, beta and whole frequency bands showed an increase after the induction of anesthesia. Conversely, the alpha band showed a large decrease after LOC. The total PSS of the delta and the whole frequency bands increased after LOC, while it significantly decreased for the other bands. The total PSS of the whole frequency band changed in the opposite direction of the genuine PSS after LOC.

## Discussion

The major findings of this study are (1) our measure of genuine PSS reflects true phase synchronization, as demonstrated by the N-Tori model, (2) spurious PSS significantly increases during general anesthesia, (3) the most influential factor for genuine PSS is the EEG reference while the most influential factor for spurious PSS is the low frequency spectra, and (4) there were individual temporal patterns for genuine and spurious PSS in each frequency band during general anesthesia.

### Spurious PSS Increases as Frequency Decreases in Simulated Data

The simulated model data clearly demonstrates the potential problem with conventional mean phase coherence by revealing spurious phase coherence for a time series with periods of zero true coherence. The problem is exacerbated when simulated frequencies are decreased and resolves as frequency increases. Significant spurious PSS began to appear from the mean frequency below about 6 Hz, which corresponds to the theta and delta rhythms of EEG. Consequently, our data suggest that, for an EEG that has a dominant frequency below the theta rhythm, spurious component measures should be taken into account.

### Change of Spectral Content during General Anesthesia is Associated with Increased Spurious PSS

The analysis of randomized EEG data acquired during anesthesia demonstrated the effect of spectral content on the three PSS indices (Figure 3). The spurious PSS was significantly increased after LOC, in which the lower frequency spectral content dominates (Figure 2a). The spurious PSS gradually returned to the level of the baseline state in association with the returned spectral contents after ROC. As expected, the genuine PSS for randomized data had nearly zero values and the total PSS had the same pattern of temporal change as that of the spurious PSS. This result clearly demonstrates the potential unreliability of conventional coherence measures (nonlinear phase synchronization and linear Pearson correlation coefficient) without monitoring the finite size effect of the EEG during anesthesia. This would also hold true for analyses of non-stationary physiological data such as epilepsy, sleep and pharmacologically-induced states. Considering the numerous uses of both coherence measures in brain network analysis, careful interpretation is necessary.

### Factors Affecting Genuine and Spurious PSS

We investigated which factor among the data length, type of EEG reference and frequency bands amplifies the finite size effect. Genuine PSS depends on the type of EEG reference and the frequency band of EEG. The unipolar EEG reference (A2) had the largest genuine PSS compared to those of the other EEG references. This may be due to the common contribution of a reference’s fluctuation to all of the EEG channels. For genuine PSS, there was no tendency of linear increases or decreases over different frequency bands. For spurious PSS, the frequency band was the most influential factor in comparison with the changes induced by the EEG reference and window size. Lower frequency bands produced more spurious phase synchronization in EEG (in Figure 4: delta, theta and alpha >> beta, gamma and whole). The two types of bipolar EEGs and common average reference had a similar level of spurious PSS. Consequently, the most influential factors on the finite size effect were different for genuine and spurious PSS. Among the examined factors, the EEG reference was the most influential factor for the genuine PSS, whereas the power spectrum was the most influential for the spurious PSS. The window size, tested from 5 seconds to 60 seconds, had no significant influence on either genuine or spurious PSS estimations. Although a small window size produces less robust PSS estimation, the large size of an ensemble (for instance, 147 windows for 5 seconds) seems to reduce the variance of the PSS estimations. The significant influence of the EEG reference on the calculation of phase synchronization has been discussed in several studies [12]–[14], but its effect on the genuine and spurious phase synchronization components under various conditions was a novel aspect of our study. However, our measure of genuine PSS could not be the direct solution for the fundamental measurement problems of EEG such as volume conduction, which gives rise to a larger coherence for EEG recording. One way to reduce the volume conduction effect in the PSS indices would be to use a coherence measure that is relatively robust for volume conduction, instead of using mean phase synchronization and Pearson correlation coefficient. For instance, phase lag index [26] or weighted phase lag index [27] could be potential candidates., The PSS indices presented in this study will be useful for optimizing coherence measurements and the simultaneous measurement of genuine and spurious PSS can help prevent incorrect interpretations of phase synchronization structure for a given EEG dataset.

### Patterns of the three PSS Indices during General Anesthesia

The genuine, spurious and total PSS evolved with typical temporal patterns corresponding to the states of consciousness and different frequency bands during general anesthesia (Fig.5a–f). After LOC, the genuine PSS decreased in most frequency bands. The decreased level of genuine PSS for the alpha band persisted until the end of the experiment, while for the other frequency bands it returned to the baseline during the anesthetized state (∼ 1 minute). It is also of interest to note that the mean values of genuine PSS for the gamma band corresponded to the subject’s behavioral responses by remaining decreased during the unconscious state and increasing in association with behavioral responsiveness. A notable difference between the alpha and gamma bands is that the decreased level of genuine PSS persisted after ROC in the alpha band, while it returned to the baseline level in the gamma band. Therefore, these typical temporal patterns of genuine PSS for different frequency bands seem to reveal diverse functional mechanisms for modulating phase synchronization–and possibly information integration–during general anesthesia. The reduced genuine PSS is consistent with the loss of functional connectivity in the brain during unconscious state [4] [28]–[30]. Otherwise, the largely increased spurious PSS in the whole frequency band demonstrated the potential risk for inaccurate determination of functional connectivity during unconscious states.

The spurious PSS also showed diverse temporal evolution patterns depending on the state of consciousness and the frequency band. After LOC, the spurious PSS was significantly increased in the delta, beta and whole frequency bands. Conversely, the spurious PSS of the alpha band EEG significantly decreased in unconscious state. The spurious PSS had a distinct temporal evolution pattern corresponding to each frequency band: increased and returned (delta and whole), unchanged (theta and gamma), decreased and then unchanged (alpha), gradually increased (beta).

### Limitations

This study has a number of limitations. The genuine PSS may not control for all possible factors that can generate spurious elements. In particular, the spurious PSS mainly focuses on the random phase synchronization components generated by spectral content. It does not, however, consider the volume conduction effect at all. We therefore use the term “genuine” with respect to PSS based on previously published terminology and acknowledge that it may not reflect true PSS with perfect accuracy. Furthermore, in order to normalize the total, spurious and genuine coherence components, the three PSS indices were divided with different denominators. Thus, the PSS indices represent relative rather than absolute genuine or spurious coherence for a given EEG dataset. The total PSS is therefore not exactly the sum of genuine and spurious PSS. Another limitation is that the N-tori model cannot represent the hierarchical functional structure of the brain; thus, a more sophisticated model is needed for realistic anesthesia EEG simulation [31]. We demonstrated the validity of PSS indices with the model data, but for EEG recorded during anesthesia we assumed the consistency of volume conduction across states.

### Conclusion

In conclusion, our proposed measures of genuine and spurious PSS can clarify the interpretations of phase synchronization structure for a given EEG dataset. Genuine and spurious PSS are associated with complex temporal evolution patterns depending on the state of consciousness. Because of significant spurious phase synchronization, simultaneous monitoring of genuine and spurious PSS is necessary. This approach may also be beneficial in elucidating true functional connectivity based on coherence measures for non-stationary physiological data in which very low frequency spectra dominate.

## Materials and Methods

The Institutional Review Board (IRB) of Asan Medical Center approved this study in human volunteers. After IRB approval and written informed consent, ten normal human subjects were studied on two separate occasions with 21-channel EEG. Three states were investigated: 1) baseline consciousness, 2) general anesthesia, defined as loss of response to a command after 2 mg/kg propofol, and 3) recovery, defined as return of responsiveness. The EEG data were originally gathered and analyzed using different methods for a study of the frontoparietal system; full details of anesthetic protocol can be found in [3].

The EEGs of 21 channels (Fp1, Fp2, F3, F4, F5, F6, F7, F8, Fz, C3, C4, Cz, T7, T8, P3, P4, P5, P6, P7, P8, Pz referenced by A2, 10–20 system) were recorded on the bed with closed eyes, with a sampling frequency of 256 Hz and 16-bits analog-to-digital precision by WEEG-32® (LXE3232-RF, Laxtha Inc., Daejeon, Korea). Baseline EEG was recorded for 5 min before an intravenous bolus of propofol. EEG was recorded continuously during and after the intravenous bolus of propofol, and up to 10 min after ROC. For the band pass filtering we used the fourth order Butterworth filter to avoid a possible shifting of the signal phases (in Matlab Signal Processing Toolbox).

### Estimation of Genuine, Spurious and Total Phase Synchronization Strengths

The genuine, spurious and total PSS were defined based on the decomposition of spatial synchronization structures of multi-channel EEG data and the comparison with its randomized data set. The decomposition of a phase synchronization matrix *S* into principle components reduces the number of entries from *M*(*M*−1)/2 → *M*, where *M* is the number of EEG channels. This simplifies the problem compared to using all entries of phase synchronization matrix *S.* By using principle components, we were also able to detect a typical coherence structure of multivariate data such as an “eigenvalue repulsion” in which the smallest principle component could produce statistically more significant information than that of the largest eigenvalue [18]. In other words, the local components contain important information about the correlation structure of a multivariate dataset; therefore, considering each local component is important.

The phases 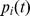 of an EEG channel 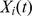 were defined by the Hilbert transform method, and the mean phase coherence 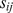
between two phase sequences 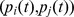 was calculated with the average of phase differences [32]. The entries of phase synchronization matrix *S* were constructed by the mean phase coherences of all possible pairs of EEG channels. The singular value decomposition of matrix S produces eigenvalues 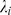 and eigenvectors 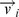:
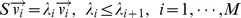

1. If all channel data are “completely independent,” all non-diagonal elements of *S* will tend to zero if the length of data *T→∞.* Then, all eigenvalues 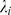
are 1.
2. For any finite *T*, the non-diagonal elements 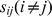
have non-zero values. The non-zero values are randomly distributed around unity, even in the case of completely independent data. The width of the distribution is determined by the length of data *T* and the frequency contents of the signals, therefore, the width associates with spurious PSS [13] [20].
3. If all 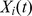
are “identical”, only one nonzero eigenvalue exists, while the other eigenvalues are zero. 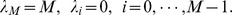

These properties were used to evaluate how much a given EEG data set deviates from completely independent or identical data (the property (1) and (3)). With the property (2), we can estimate the spurious PSS.

Randomized data sets that have the same spectral contents and amplitude distribution, but without the phase coherence between signals, were used for estimating the spurious PSS [33]. All eigenvalues for random data are supposed to be 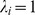 by the property (1). The non-zero eigenvalues (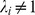) indicate the random coherence generated for a given EEG data. The spurious phase synchronization depends on the combination of a specific spectral content and a specific length data.

a Genuine PSS quantifies the phase synchronization that deviates from that of the randomized data set [34]. Genuine PSS is defined as the fraction of the difference between the original and randomized EEG to the difference between completely correlated data and randomized EEG,
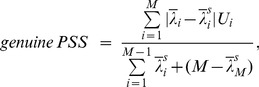
(1)where 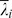 and 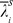 denote the averages of the ith eigenvalue for the original data set and the randomized data set, respectively. The significant deviation was determined with the Mann-Whitney Wilcoxon U-test. If the null hypothesis for equal means 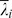
and 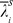
was rejected with p<0.05, then 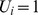, otherwise, 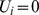. If the average eigenvalues of the original data are not significantly deviated from those of the randomized data, the numerator has a zero value. On the other hand, if all phases are identical, then the largest eigenvalue 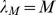
and the others equal zero. Since the numerator and denominator (normalization factor) are identical, the genuine PSS is 1. Therefore, the genuine PSS has a value between 0 and 1.
b Spurious PSS quantifies the spurious phase synchronization produced by finite size and certain spectral contents of the data. Spurious PSS is defined as the fraction of the difference between independent data and randomized EEG to the difference between independent data and completely correlated data,

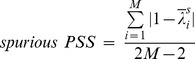
(2)Since the randomized data are uncorrelated across channels, 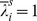
for all *i* according to the property (1). If 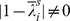, it is a spurious component produced by the specific combination of a data length *T* and spectral content.

c Total PSS measures the amount of total phase synchronization strength (spurious and genuine PSS) contained in a given set of data. Total PSS is defined as the fraction of the difference between independent data and original EEG to the difference between independent data and completely correlated data,

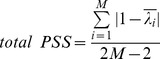
(3)The total PSS quantifies the deviation of the eigenvalues of a given dataset from those of the ideal case (completely uncorrelated and *T→∞*). Since the given dataset may contain genuine and spurious phase synchronization components, the difference from the ideally uncorrelated case estimates the amount of spurious and genuine phase synchronization strengths for a given dataset. For (b) and (c), the denominator *2 M−2* is the normalization factor. For the completely uncorrelated case the numerator is 0, while for completely correlated case the numerator 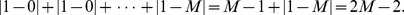 Therefore, the genuine PSS, spurious PSS and total PSS have a value within 0 and 1, respectively. See Table 2 for a glossary of terms.

**Table 2:** Glossary.

Term	Definition
**Spurious PSS**	Estimation of spurious elements produced by a specific spectral content in a finite data length in the phase synchronization for a given two EEG signals.
**Genuine PSS**	Evaluation of how much the phase synchronization of a given EEG signal deviates from the estimated spurious PSS.
**Total PSS**	Estimation of the amount of total phase synchronization (spurious and genuine PSS) between two EEG signals.
**Eigenvalue/eigenvector**	These concepts are used in linear algebra, representing the properties of a matrix. In a diagonalized matrix, the eigenvalues are the numbers on the diagonal and the eigenvectors are the basis vector to which these numbers refer. This enables the analysis of a given matrix in a way similar to a diagonal matrix, which simplifies the process. In a diagonalized coherence matrix, we can more easily determine the principle coherence elements with large eigenvalues.
**Randomized (surrogate) data**	A replica of a given EEG signal that retains the original spectral contents but with randomized phases.
**Ensemble average**	A given time series is replicated many times over in order to generate an enormous number of copies. The replica is allowed to differ microscopically in the time series, while retaining the same general properties (such as spectral content). Such a collection of replicated time series is called an ensemble, and the average is called an ensemble average.

Definitions of terminology used. PSS = phase synchronization strength, EEG = electroencephalogram.

In the equations (1), (2) and (3), the PSS indices were estimated based on the ensemble average from segmented small windows. In the equations (1), (2) and (3), a 10 second-long EEG epoch was segmented into smaller windows (2 seconds). The average eigenvalue 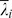 for an EEG epoch (*i*) was estimated from the ensemble of small windows. For 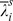, 20 randomized EEG datasets were generated for the original EEG epoch and segmented into smaller windows in the same way. The randomized data set has the same power spectrum and amplitude distribution of the original data [33]. This ensemble average reduces the variability in the estimation of phase synchronization for a given EEG epoch.

### Testing the Performance of this Method with Simulated EEG Data

The validity of the genuine and spurious PSS was tested with multivariate model data, in which the mean frequency and the phase coherence were modulated. We hypothesized that lower mean frequency produces higher spurious PSS and that genuine PSS correlates well with the given phase coherence. With the simulated EEG time series, we investigated the effects of mean frequency, phase coherence and their combination on the spurious, genuine and total PSS. Twenty time series were generated from N-tori [35]:
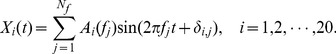
(4)


The amplitudes 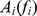 was adjusted with the Wigner distribution [36]:
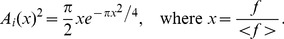
(5)


A time series, 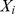 is the superposition of 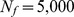 sine waves with randomly chosen frequencies 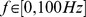. The mean frequency, 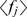, was set to be 10 Hz in the first half of the period, and it was linearly shifted to 1 Hz in the second half to see the effect of mean frequency on the PSS indices. However, the total power was kept constant over all time series and throughout a time series. The phase coherence between time series was determined by the partial phase of sine wave 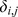. For no phase coherency between time series, the partial phases 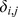 for each time series 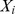
was chosen from a uniform distribution ranging from 0 to 2π. On the other hand, for a phase coherency between time series, the partial phase 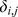 for each time series was chosen from a normal distribution with mean phase (i.e., 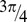 ). The strength of phase coherence was modulated with the standard deviation of phase distribution: a smaller standard deviation produces stronger phase coherence. The sampling frequency was 200 Hz in this simulated dataset. The mean frequency and the phase coherence of the time series were modulated in different ways in seven time periods (See Table 3).

**Table 3:** The dominant frequency and phase coherence of the seven periods in the simulation data.

**Time period (second)**	0–20	20–40	40–60	60–80	80–100	100–120	120–140
**Phase distribution**	U	U→N	N	N	N	N→U	U
**Dominant frequency (Hz)**	10	10	10	10→1	1	1	1
**Expected genuine/spurious Phase coherence**	0/↔	⇑/↔	↑/↔	↑/⇑	↑/↑	⇓/↑	0/↑

‘U’ indicates the uniform distribution of phases (0–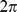
_)_, and ‘N’ indicates the normal distribution of mean of 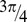 and standard deviation of 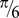. U→N denotes the linear transition from the uniform distribution to the normal distribution, and vice versa for N→U. ‘↔’ denotes a maintained value. ‘↑’ denotes an increased value. ‘⇑’ denotes a linearly increasing value. ‘⇓’ is vice versa.

1. The first period (0–20 s): the phases 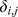 were set to be uniformly distributed (0–2π) and 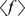
is 10 Hz. Thus the time series are independent. Zero genuine PSS but non-zero spurious PSS were expected.
2. The second period (20–40 s): the distribution of phases was transformed from the uniform distribution to the normal distribution with mean of 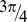 and standard deviation of 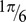. Thus, the phase coherence was gradually increasing. A gradually increasing genuine PSS was expected.
3. The third period (40–60 s): the same normal distribution of phases was given. Therefore, the phase coherence is maintained at the same level. No changes were expected in genuine and spurious PSS.
4. The fourth period (60–80 s): Using the normal distribution of phases, the mean frequency 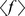 was shifted from 10 Hz to 1 Hz. A gradually increasing spurious PSS was expected.
5. The fifth period (80–100 s): the same normal distribution of phases and the mean frequency 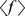 = 1 Hz are used. No changes were expected in genuine and spurious PSS.
6. The sixth period (100–120 s): the distribution of phases is transformed from the normal distribution to the uniform distribution, while 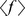 = 1 Hz. The phase coherence is gradually decreasing. Thus, a gradually decreasing genuine PSS was expected.
7. The seventh period (120–140 s): the uniform distribution of phases is maintained, while 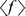 = 1 Hz. No changes were expected in genuine and spurious PSS.

### Application to EEG during General Anesthesia

The PSS indices were applied to anesthesia EEG data, which consisted of three different states: baseline conscious state, unconscious state (loss of consciousness, LOC) and recovery state (return of consciousness, ROC). Each state consisted of a 5 minute-long EEG epoch. The moving window method was used to investigate the temporal evolution patterns of the genuine, spurious and total PSS during general anesthesia. The window size of 10 seconds without overlap was used to achieve the appropriate time resolution for the fast state transitions during general anesthesia, which take place in a short term after LOC and before ROC. To obtain an ensemble of phase synchronization matrices in the equation (1), (2) and (3), the 10 second-long windows were segmented into five sub-windows (2 second long windows without overlapping). Thus, for a 10 second-long EEG window, 5 sub-windows of original EEG data and 100 ( = 5×20) sub-windows of 20 randomized data sets were generated. The mean and standard deviation of eigenvalues were calculated with these ensembles of sub-windows for the original EEG and randomized EEG data sets. The 2 second-long sub-window was determined as the smallest among various sizes that gave similar PSS indices as larger sub-windows tested. The 10 second long window was determined by a trade-off of the best time resolution to assess the temporal pattern of state transitions and the robustness of the PSS results, which was accomplished by searching various sizes of the window (10 to 30 seconds). In addition, since anesthesia EEG is highly non-stationary, this ensemble average with sub-windows would reduce the variability in the estimation of PSS indices.

### Assessing the Role of EEG References

The effect of EEG reference on the genuine, spurious and total PSS indices was studied with four different types of EEG references (unipolar, common average, longitudinal bipolar and transverse bipolar). For the unipolar EEG reference, the A2 channel was used as the referential electrode; for the common average EEG reference, the averaged EEG over 21 EEG channels at each time *t* was used as the reference. For the longitudinal bipolar EEG reference, (Fp1-F7, F7-T7, T7-P7, Fp1-F5, F5-C3, Fp2-F6, F4-C4, F8-T8, CZ-P4, Fz-Cz, Fp1-F3, F3-C3, C3-P3, Fp2-F4, F6-C4, T8-P8, C4-P6, Cz-Pz:18 longitudinal bipolar montage) were used. For the transverse bipolar EEG reference, (FP1-Fp2, Fp2-F8, F7-F5, F5-F3, F3-Fz, Fz-F4, F4-F6, F6-F8, T7-C3, C3-Cz, Cz-C4, C4-T8, P7-P5, P5-P3, P3-P4, Pz-P4, P4-P6, P6-P8∶18 transverse bipolar montage) were used. The referential montages were chosen according to the standard electrode montage of the American Clinical Neurophysiology Society [37]. Since O1 and O2 channels were not recorded in this study, we used a modified referential montage without O1 and O2.

### Statistical Analysis

The moving window analysis was not well-suited to a repeated measures one-way ANOVA because of the many repeated measurements (∼ 30 windows). Thus, to apply the conventional ANOVA and post-hoc analysis, each state (consciousness, anesthesia, recovery) was separated into two sub-states. The Baseline conscious state was separated into B1 (from 0 to 2.5 minutes) and B2 (from 2.5 minutes to 5 minutes), the Anesthetized state was separated into A1 (0 to 2.5 minutes after LOC) and A2 (−2.5 to 0 minutes before ROC) and the Recovery state was separated into R1 (0 to 2.5 minutes after ROC) and R2 (2.5 to 5 minutes after ROC). Since the duration of the anesthetized state is different for each subject, we separated the sub-states based on the LOC and ROC times in order to facilitate statistical comparison. The three PSS indices were compared across the six sub-states; the significance was assessed by a repeated measures one-way ANOVA and a *post hoc* analysis using Tukey’s multi-comparison test. A *p* value <0.05 was considered significant. The GraphPad Prism Version 5.0c (GraphPad Software Inc. San Diego CA) was used.
